# Nutrition as the Foundation of Human Capital: Pathways to Holistic Development

**DOI:** 10.1111/mcn.70196

**Published:** 2026-05-21

**Authors:** Demeke Mekonnen Hassen

**Affiliations:** ^1^ Founder and Chairman, YADAM Foundation Addis Ababa Ethiopia

## Abstract

Human capital is widely recognized as central to economic growth and social progress. However, commonly used development measurements often assess outcomes achieved in adolescence or adulthood. These approaches overlook the formative biological processes that shape lifelong capability. In line with this, this Perspective argues that maternal and early childhood nutrition, particularly during the first 1000 days, constitutes the critical foundation of human capital formation. During this critical window, rapid brain development, immune programming, and metabolic adaptation occur, making nutritional adequacy, quality, and diversity essential for cognitive development, executive function, and long‐term productivity. Human capital development during the first 1000 days—rooted in maternal and early‐life nutrition and identified as the Creation phase in this Perspective—is considered a necessary condition for investments in education and skill formation in later life stages. Despite this, widely used development measures, including the human development index (HDI), remain largely retrospective in approach and output‐oriented in focus. The Perspective proposes an adjustment to the HDI by incorporating maternal, birth, child nutrition, and early developmental indicators to better capture the biological foundations of capability. This approach would create an opportunity to ensure multisectoral accountability, address intergenerational and geographic inequities, and align policy priorities with the origins of human capital and development.

## Summary

- Nutrition during the first 1000 days provides the biological infrastructure for human capital, directly influencing neurodevelopment, cognitive function, and lifelong productivity.
- Existing metrics like the human development index (HDI) are output‐oriented; they capture the outcomes achieved but fail to integrate the formative biological processes that enable them.
- A nutrition‐adjusted HDI (NHDI) is proposed to integrate maternal and child nutrition and early development indicators, shifting development measurement from purely economic or survival outcomes to a process‐based evaluation of human capability.
- Integrating early‐life nutrition into development metrics can strengthen policy prioritization, accountability, and equity.

## 1 Introduction

Human capital has long been regarded as central to economic growth and social progress (Becker 1964; Schultz 1961). Accordingly, investments in education, skills, and health are widely recognized as drivers of labor productivity, resilience, and national prosperity. A substantial body of interdisciplinary evidence also demonstrates that skill formation is dynamic and cumulative in nature: early inputs tend to shape the productivity of later investments—skills beget skills, and deficits during childhood are more likely to be difficult and costly to reverse (Heckman 2006; Cunha and Heckman 2007). Human capabilities are also formed through a multistage process often defined in space (Holla et al. 2026) and time (Heckman 2008). In these spatio‐temporal processes, the biological, cognitive, and socio‐emotional bases are laid early in life to condition subsequent learning and adaptation.

Within this process, the first 1000 days have emerged as a uniquely sensitive period and a critical window of opportunity for shaping individuals' lifelong trajectories. It is in this period that the brain grows rapidly, the immune system is programmed, and metabolic development occurs. These critical developments make the adequacy, quality, and diversity of nutrition during pregnancy and early childhood influence not only survival but also the architecture of the developing brain, affecting cognition, executive function, emotional regulation, and long‐term health (Clark et al. 2020; Black et al. 2015).

However, despite the robust evidence, current human development metrics largely measure outcomes achieved in adolescence or adulthood rather than the formative biological conditions that make those outcomes possible. The human development index (HDI), for example, captures life expectancy, years of schooling, and income per capita. These are critical indicators, but they are retrospective in nature. They reflect accumulated achievements, not the early‐life processes that enabled them.

This Perspective argues that maternal and early childhood nutrition are not peripheral health concerns but critical drivers of human capital formation and human development. Development metrics that omit early‐life nutritional conditions risk systematically mismeasuring long‐term national economic and social progress. A nutrition‐sensitive adjustment to human development measurement is therefore both conceptually and practically necessary.

The remainder of this Perspective is structured as follows. Section 2 examines the biological foundations of human capital during the first 1000 days. Section 3 explores intergenerational transmission and maternal influences. Section 4 situates these dynamics within changing global contexts. Section 5 critically assesses gaps in current development metrics. Section 6 proposes a nutrition‐responsive adjustment to the HDI. Section 7 discusses policy and equity implications, followed by concluding reflections in Section 8.

## 2 The First 1000 Days: Biological Infrastructure of Human Capital

Human capital does not begin in classrooms or labor markets; it begins in utero and at home. Studies have demonstrated that the prenatal environment significantly shapes fetal growth, organ development, and brain formation (Almond and Currie 2011; Fitzgerald et al. 2020). The brain, for instance, undergoes rapid synaptogenesis and myelination, and by the second year of a child's life, 80% of brain formation is complete (Dekaban and Sadowsky 1978). During this period, neural circuits governing language, memory, executive function, and socio‐emotional regulation are formed at extraordinary speed. This process requires adequate energy, high‐quality protein, essential fatty acids, and micronutrients such as iron, iodine, zinc, and DHA. Related studies also show that maternal undernutrition, anemia, and micronutrient deficiencies during this time increase the risk of intrauterine growth restriction and low birth weight (Fall et al. 2003; Asferie et al. 2025). For example, iron deficiency during infancy can alter myelination and neurotransmitter function, affecting attention and memory. Iodine deficiency impairs thyroid hormone production, which is essential for brain development. Zinc and DHA play critical roles in synaptic plasticity. These conditions are associated with impaired cognitive development, reduced immune competence, and heightened vulnerability to chronic disease across the life course (Black et al. 2013).

Chronic undernutrition, particularly stunting, is also linked to structural and functional alterations in the developing brain. Neuroimaging studies have demonstrated differences in brain volumetry and visual working memory among children exposed to early nutritional deprivation (Wijeakumar et al. 2023; Koshy et al. 2025). These biological disruptions manifest in lower cognitive test scores, weaker executive function, and reduced school readiness. Critically, these early effects are not limited to childhood. These biological markers of early cognitive outcomes represent additional dimensions of human capital distinct from the longevity metrics used in the current HDI. These metrics can serve as leading indicators of cognitive potential and economic productivity. By incorporating these leading indicators into the standard HDI, development measurement can move beyond the duration of life to capture the formative quality of the cognitive assets individuals possess.

Longitudinal and randomized controlled trial (RCT) studies have also demonstrated that improved nutritional supplementation early in life is associated with increased schooling attainment and higher adult wages later in labor markets. Conversely, childhood stunting results in fewer years of educational attainment, lower academic achievement, and reduced lifetime earnings and labor productivity (Dewey and Begum 2011; Alderman 2006; Soliman et al. 2021). Empirical studies confirm these causal linkages. For example, a longitudinal study from rural Zimbabwe finds that stunted preschool children had 0.85 fewer years of schooling, leading to a 14% reduction in lifetime earnings compared to what would have been possible (Alderman 2006). (Woldehanna et al. 2018) also find that stunted children in Ethiopia scored 16.1% less in the Peabody Picture Vocabulary Test and 48.8% less in the Quantitative Assessment test at the age of eight. In contrast, a long‐term RCT‐based study from Guatemala shows that children who received a high‐protein nutritional supplement before the age of three earned 46% higher wages in adulthood than their peers (Hoddinott et al. 2008). These findings emphasize a critical point: early nutrition influences not merely health status but the quality and productivity of human capital.

Overall, while the empirical evidence linking early nutrition to cognitive development and outcomes later in life is substantial, it is important to note that these relationships are shaped by complex and interacting pathways. Because much of the available empirical evidence, including that presented above, comes from observational studies, quasi‐experimental designs, and randomized interventions, the extent of the causal effects and implications may vary across countries and communities. In this regard, it is widely understood that nutritional influences operate alongside broader structural factors, including poverty, education systems, gender norms, and environmental conditions. This Perspective therefore interprets early nutrition as a critical, though not exclusive, determinant within a wider system shaping human capital formation and human development. It lays the biological basis upon which long‐term outcomes are built. Without this, later investments in education, health, and skills training yield suboptimal returns.

## 3 Maternal Nutrition and Intergenerational Transmission

Human capital formation is inherently intergenerational. Nutritional deficiencies may begin prenatally when brain development begins (Black et al. 2015). This means that a mother's nutritional status can influence her child's birth outcomes, growth trajectory, and developmental potential. As discussed earlier, maternal undernutrition increases the likelihood of low birth weight, which is associated with delayed cognitive development and increased risk of chronic disease in adulthood. Maternal anemia affects oxygen transport to the fetus, potentially impairing brain development. Short maternal stature, often a consequence of childhood undernutrition, is also associated with an increased risk of obstructed labor and poor birth outcomes (Victora et al. 2021).

**Figure 1:**
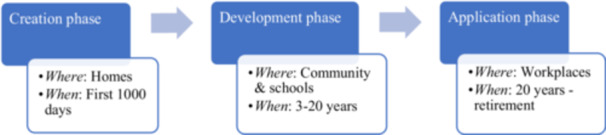
Human capital development path and the environment. *Note:* The place concepts embedded in this conceptualization are informed by evidence in the recent human capital report (Holla et al. 2026). Source: Own conceptualization.

To better explain the temporal and intergenerational nature of deprivation—both within an individual's life course and between generations—and the overarching and critical role of nutrition in various space‐settings (Holla et al. 2026), we can conceptualize human capital formation into three interlinked, and to some extent overlapping and non‐linear, phases: Creation, Development, and Application (Figure 1). These development phases shape the link between biological potential and the surrounding environment, such as homes, communities, schools, and workplaces. Viewed as an interconnected system, these phases create both reinforcing and constraining loops. In this regard, a robust Creation phase could be amplified or undermined by the Development phase due to factors such as quality of education, market access, WASH, and health. The extent to which these, in turn, influence long‐term outcomes is also moderated by labor market dynamics during the Application phase. In this Perspective, it is argued that while the Creation phase is the foundational and strongly conditioning factor for human potential and holistic development, the later phases could strongly determine whether that early‐life potential is harvested or wasted. These phases also help to give due emphasis to the places where human capital is created. Accordingly, while the Creation phase highlights the critical role of the home environment, the Development and Application phases point to the importance of communities, schools, and workplaces (Holla et al. 2026). For example, studies indicate that while responsive feeding by mothers at home can improve children's weight and developmental outcomes through better feeding practices (Pérez‐Escamilla et al. 2019), the outcomes can be moderated by the availability and quality of handwashing and sanitation, parenting skills, and psychosocial stimulation (Pérez‐Escamilla and Moran 2017). Growing evidence also suggests that several external factors, including social protection, influence the role of nutrition in early childhood development (Pérez‐Escamilla and Moran 2017). In this regard, the place‐based dimension of early human development underscores the need to focus not only on what and when to invest in nutrition but also where to invest, allowing for a more comprehensive understanding of the nutrition‐human capital‐development nexus.

This framework does not intend to replace existing human capital theory and frameworks, but to extend the evidence by explicitly embedding biological foundations as a necessary condition for later capital accumulation. In addition, while presented sequentially for analytical clarity, these phases are dynamic, non‐linear, and interdependent rather than strictly linear. Later interventions, including school feeding programs, nutrition supplementation, education, health services, and on‐the‐job skill training, can partially mitigate early disadvantages and deficits, although often at higher cost and with varying effectiveness. The framework is therefore intended as a heuristic to highlight the foundational role of early‐life conditions, rather than a deterministic model.

Related to this and consistent with the Creation phase, the World Health Organization, UNICEF, and the World Bank have also emphasized through the Nurturing Care Framework that optimal child development requires adequate nutrition, health services, responsive caregiving, safety, and early learning opportunities (WHO, UNICEF, and World Bank 2018; Black et al. 2021). In this framework too, nutrition interacts synergistically with these domains. That is, a well‐nourished child is more capable of benefiting from stimulation and early learning at home and school. Conversely, early nutritional deprivation constrains responsiveness, attention, and engagement, limiting the effectiveness of caregiving and educational inputs.

Breastfeeding practices further illustrate this interaction. Exclusive breastfeeding during the first 6 months provides essential nutrients and bioactive compounds that support brain development and immune protection (Cusick and Georgieff 2016). Complementary feeding practices determine whether children receive sufficient dietary diversity and nutrient density during a period of rapid growth (Maternal and Child Nutrition Study Group 2013). Inadequate complementary feeding, by contrast, can result in micronutrient deficiencies that impair neurodevelopment and immune resilience.

A substantial body of evidence also shows that when maternal and early childhood nutrition are compromised, the consequences extend beyond individual and short‐term outcomes. Reduced cognitive development can translate into lower educational attainment in school, limited employment opportunities in labor markets, and constrained income. In turn, lower maternal income and educational attainment can perpetuate inadequate nutrition for the next generation. This intergenerational cycle underscores the critical nature of early nutritional interventions not only as health measures but as structural strategies for breaking persistent poverty and inequality.

## 4 Changing Contexts and the Rising Cost of Early Nutritional Failure

We are also standing at a critical point in time where global economic, social, and technological landscapes are changing rapidly. Knowledge‐based economies increasingly value cognitive flexibility, problem‐solving, and socio‐emotional competencies. While education systems adapt to digital transformation, the underlying biological capacities required for learning remain rooted in early neurodevelopment (Luckin and Holmes 2016; Bin Rashid and Kausik 2024).

In such contexts, the cost of early nutritional deprivation may become even more pronounced. Children who enter school with compromised executive function or language development face cumulative disadvantages. Even with access to schooling, their ability to absorb and apply knowledge may be constrained by early biological deficits. As labor markets demand adaptive skills and lifelong learning, early cognitive impairments can translate into enduring economic exclusion. In this changing landscape, the call is clear and timely: nutrition must be recognized as a dynamic determinant of human agency and economic participation. Development measurement systems must also evolve accordingly, from measuring outcome decades later to capturing critical signals and development processes at early stage.

## 5 The Measurement Gaps in Human Development

The HDI was one of the transformative innovations in economic science, shifting attention from income alone to a multidimensional conception of development. By incorporating life expectancy, education, and income, it broadened policy discourse (ul Haq 1995). However, it remains fundamentally output‐oriented. It measures achievements after decades of accumulated experiences.

Other limitations of the HDI stem from how its components, such as Gross Domestic Product (GDP), are designed to measure. GDP, for example, systematically undervalues or excludes foundational human development inputs—such as breastfeeding. While breastfeeding, an activity with well‐established benefits for child development and long‐term health, is not counted in GDP, the purchase of commercial infant milk formula contributes positively to economic output (Baker et al. 2023). This creates a paradox where improvements in optimal child feeding practices may not be reflected in, and may even reduce, measured economic performance due to the exclusion of unpaid caregiving—largely provided by women—from GDP measurement (Baker et al. 2023). These inconsistencies highlight the need for development metrics that better capture the true foundations of human capability. As a result, gross national income (GNI) per capita which represens standard of living in HDI, does not reveal whether individuals entered adulthood with optimal biological assets. Moreover, while life expectancy reflects survival but does not capture the quality of early brain development, years of schooling measure the duration of attendance, not cognitive readiness or executive function.

In this regard, it is important to acknowledge that existing frameworks have begun to incorporate elements of early‐life development. The World Bank's Human Capital Index (HCI), for instance, includes stunting as a proxy for early childhood health and development (World Bank 2020), while UNICEF's Early Childhood Development Index (ECDI) captures aspects of cognitive, physical, and socio‐emotional readiness (UNICEF 2023). These represent important advances. However, they differ in purpose, age coverage, and influence from the HDI. The HCI is primarily designed as a forward‐looking productivity measure linked to future earnings, and the ECDI focuses on child development outcomes at a specific life stage (24–59 months). In contrast, the HDI remains the most widely recognized and politically salient composite measure of human development, shaping global discourse and national benchmarking. The contribution of this Perspective is therefore not to replicate existing indices, but to extend the conceptual foundation of the HDI by explicitly integrating early‐life nutritional conditions as core determinants of capability formation. This shifts the metric from a purely outcome‐based framework toward one that also reflects foundational processes.

In the past, various extensions were made to the HDI to address inequality, gender disparities, and environmental pressures. Yet early‐life nutritional conditions, mainly during the first 1000 days, remain largely peripheral. Indicators such as stunting or low birth weight are typically treated as health statistics rather than core components of development measurement. This omission creates a conceptual inconsistency. The central argument of this Perspective in this regard is that if human development aims to expand capabilities, then the formative biological determinants of those capabilities warrant central attention. Thus, measuring adult achievements without accounting for early‐life determinants risks misinterpreting both progress and stagnation.

## 6 Toward a Nutrition‐Responsive Human Development Index

In light of the above gaps and needs, this Perspective proposes an adjustment to the HDI to incorporate early‐life nutrition conditions, which could be termed the nutrition‐adjusted HDI (NHDI). This approach could address the existing gap by incorporating child nutritional indicators as a foundational dimension of human development. Such an index may include four dimensions: maternal and birth indicators, child nutrition outcomes, nutrition‐sensitive inputs, and early developmental indicators. Maternal and birth indicators could include the prevalence of low birth weight, maternal anemia prevalence, and adolescent pregnancy rates. While child nutrition outcomes include stunting and wasting, nutrition‐sensitive inputs include (exclusive) breastfeeding, minimum acceptable diet (MAD), and minimum dietary diversity (MDD) among young children. Lastly, early developmental indicators could constitute measures of cognitive and socio‐emotional readiness at school entry. Importantly, data for most of these proposed indicators are collected through standard Demographic and Health Surveys (DHS), Multiple Indicator Cluster Surveys (MICS), and national nutrition surveillance systems.

Indicators would be normalized using established HDI methods, aggregated into a nutrition dimension index, and incorporated multiplicatively to preserve complementarity. This ensures that these indicators serve as a complement and early evidence of HDI, rather than replacing existing metrics. Their inclusion would shift development measurement toward more formative and predictive metrics.

Concerns about overlap with life expectancy and education indicators are valid. Nutritional status influences both survival and schooling attainment. However, this overlap reflects causal pathways and is temporally linked, not redundancy. Nutrition functions as a leading indicator and formative moderator of later outcomes. Including it explicitly clarifies these pathways and strengthens the interpretive power of development metrics.

Despite its immense potential advantages, integrating early‐life nutrition into the HDI is also likely to present challenges. The availability of nationally representative data in a timely manner, as well as comparability across countries, may limit consistent measurement. There is also a risk that governments could face incentives to selectively prioritize measurable and observable indicators for immediate gains over broader systemic improvements. Additionally, expanding the HDI may increase its complexity, requiring a clear methodological approach to ensure transparency and communication. These considerations underscore the importance of careful indicator selection, piloting for gradual expansion, robust data systems, and complementary consultative and qualitative assessments.

## 7 Policy and Equity Implications

Embedding nutrition within development metrics would have significant policy implications. First, it would elevate maternal and child nutrition from sectoral health issues to strategic development priorities. Investments in preconception care, micronutrient supplementation, food security, and adolescent nutrition would be recognized as central to national human capital formation. Second, it would reinforce the urgency of the first 1000 days. Governments and development partners could use the evidence to identify geographic areas and socioeconomic disparities in early‐life conditions to promote targeted interventions. Third, it would highlight intergenerational inequities. Regions with persistent maternal undernutrition and high stunting prevalence would be visibly disadvantaged in development rankings. This creates incentives for sustained and place‐based investment. It is widely understood that what we measure can shape policy prioritization. Making the role of maternal and early nutrition in human development visible to policymakers provides a stronger basis for accountability, strategic alignment, and resource allocation.

The adoption of NHDI would also involve important political economy considerations. Changes to widely used global metrics may face resistance due to concerns about comparability over time and shifts in country rankings. There are also potential equity implications, as countries or regions with historically high levels of undernutrition may appear disadvantaged, necessitating careful interpretation to avoid stigmatization. At the same time, such visibility could mobilize greater investment and international support. The interaction with global financing mechanisms, including development lending frameworks, is also critical. A nutrition‐responsive HDI (NHDI) could strengthen the case for increased investments in early childhood interventions while encouraging more integrated, multisectoral approaches to human capital development.

## 8 Conclusions

The biological and cognitive bases established during the first 1000 days shape trajectories in health, learning, and productivity later in life. Maternal and early childhood nutrition are therefore not peripheral health concerns but central determinants of human capital and national prosperity.

Existing development metrics have advanced the discourse beyond income, but they remain largely retrospective. By overlooking formative nutritional conditions, they risk mismeasuring both progress and potential.

Re‐thinking the relationship between nutrition, human capital, and human development requires integrating maternal and early‐life nutrition into the core architecture of development measurement. This calls for an adjustment of the HDI, which would represent a step toward aligning what we measure with what truly builds human capability.

Looking ahead, the proposed nutrition‐responsive adjustment to the HDI has direct relevance for the emerging post‐2030 global development agenda. As discussions evolve beyond the Sustainable Development Goals, there is a need for metrics that capture not only achieved outcomes but also the foundational processes that enable them. Integrating early‐life nutrition into development metrics would align future global human development goals with the biological origins of human capability, strengthen accountability for investments in the first 1000 days, and support more equitable and forward‐looking policy frameworks.

## Author Contributions

D.M.H. conceived the Perspective, developed and visualized the three‐phase human capital framework, conducted the literature review and synthesized it, drafted the original manuscript; and conducted all critical revisions and final editing.

## Conflicts of Interest

The author declares no conflicts of interest.

## Data Availability

This Perspective does not involve data analysis. Hence, no data is available to share.
